# Ventilator-Associated Pneumonia in Patients with COVID-19: A Systematic Review and Meta-Analysis

**DOI:** 10.3390/antibiotics10050545

**Published:** 2021-05-07

**Authors:** Mariachiara Ippolito, Giovanni Misseri, Giulia Catalisano, Claudia Marino, Giulia Ingoglia, Marta Alessi, Elisa Consiglio, Cesare Gregoretti, Antonino Giarratano, Andrea Cortegiani

**Affiliations:** 1 Department of Surgical, Oncological and Oral Science, University of Palermo, 90127 Palermo, Italy; mariachiara.ippolito@community.unipa.it (M.I.); giuliacatalisano@gmail.com (G.C.); dott.ssacmarino@gmail.com (C.M.); ingogiulia@gmail.com (G.I.); martalessi@hotmail.it (M.A.); elisa.consiglio0306@gmail.com (E.C.); cesare.gregoretti@unipa.it (C.G.); antonino.giarratano@unipa.it (A.G.); 2 Fondazione “Giglio”, 90015 Cefalù, Italy; giovannimisseri1987@gmail.com; 3 Department of Anaesthesia, Intensive Care and Emergency, Policlinico Paolo Giaccone, Via del Vespro 129, 90127 Palermo, Italy

**Keywords:** COVID-19, ventilator-associated pneumonia, meta-analysis, invasive mechanical ventilation

## Abstract

The aim of this systematic review and meta-analysis was to estimate the pooled occurrence of ventilator-associated pneumonia (VAP) among patients admitted to an intensive care unit with COVID-19 and mortality of those who developed VAP. We performed a systematic search on PubMed, EMBASE and Web of Science from inception to 2^nd^ March 2021 for nonrandomized studies specifically addressing VAP in adult patients with COVID-19 and reporting data on at least one primary outcome of interest. Random effect single-arm meta-analysis was performed for the occurrence of VAP and mortality (at the longest follow up) and ICU length of stay. Twenty studies were included in the systematic review and meta-analysis, for a total of 2611 patients with at least one episode of VAP. The pooled estimated occurrence of VAP was of 45.4% (95% C.I. 37.8–53.2%; 2611/5593 patients; I^2^ = 96%). The pooled estimated occurrence of mortality was 42.7% (95% C.I. 34–51.7%; 371/946 patients; I^2^ = 82%). The estimated summary estimated metric mean ICU LOS was 28.58 days (95% C.I. 21.4–35.8; I^2^ = 98%). Sensitivity analysis showed that patients with COVID-19 may have a higher risk of developing VAP than patients without COVID-19 (OR 3.24; 95% C.I. 2.2–4.7; P = 0.015; I^2^ = 67.7%; five studies with a comparison group).

## 1. Introduction

During the Coronavirus disease 2019 (COVID-19) pandemic, an unprecedented number of patients were admitted to intensive care units (ICUs) with COVID-19-related severe respiratory failure and underwent invasive mechanical ventilation (IMV) [1]. Invasive mechanical ventilation, especially if prolonged, is a risk factor for the occurrence of ventilator-associated pneumonia (VAP) [2,3]. Though there is no univocal definition of VAP, the most recent definitions suggest its identification by both radiological and clinical criteria, often combined with microbiological criteria [4,5,6,7]. The time span from the beginning of IMV to the fulfilment of the criteria is also an important component of the definitions [4,5,6], to exclude previously acquired pulmonary infections, thus not directly related to IMV [4]. Duration of IMV in patients with COVID-19 and admitted to ICU is often relatively long [1]. The clinical course of patients with the most severe form of COVID-19 in ICU may be complicated by the need for extracorporeal membrane oxygenation [8]. Other factors, such as the lung damage itself and the use of immunomodulant therapies (e.g., corticosteroids, anti-il-6 drugs) may also increase the risk of VAP in these cohorts of patients [9].

To date, the reported proportion of patients with COVID-19 who developed VAP COVID-19 has been variably reported. The main aim of this systematic review and meta-analysis was to estimate the pooled occurrence of VAP among patients with COVID-19 admitted to ICU and mortality of this patient population, to provide reliable data to clinicians caring for patients with COVID-19 undergoing IMV.

## 2. Materials and Methods

The protocol of this systematic review and meta-analysis was registered in Open Science Framework (https://osf.io/32mva).

We performed a systematic search of PubMed, EMBASE and Web of Science from inception to 2 March 2021 for nonrandomized studies, both prospective and retrospective, specifically addressing VAP in adult patients with COVID-19 and reporting data on at least one primary outcome of interest. Further surveillance searches were performed using the ‘related articles’ feature [10].

Primary outcomes were the occurrence of VAP and mortality at the longest available follow-up. Length of ICU stay (LOS) was an additional outcome. No language restrictions were applied to the search. Studies including less than ten patients, case reports, abstracts and not peer-reviewed articles were excluded. The search strategy included keywords as exact phrases and subject headings, according to databases syntaxes and is provided in Supplementary Material S1. All the retrieved records were independently screened from title and abstract by two authors (M.I., C.M.). The selected records were then independently reviewed from full text by the same two authors, to verify the fulfilment of the inclusion criteria. Studies were included if the screening authors agreed regarding eligibility. Disagreements at any stage were adjudicated by a third author (A.C.). The corresponding authors of the screened articles were contacted by two authors (M.I., A.C.) when questions arose regarding eligibility or data presentation at any time during this process. The reference lists of relevant articles were also searched for additional potentially relevant papers (i.e., snowballing method) by the same authors. Data extraction was performed independently by three authors (M.I., G.M., G.C.). No a priori definition of VAP was adopted, and data were extracted according to authors’ definitions. When disaggregated data were presented on multiple episodes of VAP, we extracted data on patients who had developed at least one episode of VAP, to achieve the most comprehensive estimate of the outcomes. The final version of the tabulated data was validated by all the authors involved in data collection (M.I., G.M., G.C., C.M., A.C.). The Preferred Reporting Items for Systematic reviews and Meta-Analyses (PRISMA) [11] checklists are provided as Tables S1 and S2 in Supplementary Material S2.

### 2.1. Qualitative Analysis

Two investigators (M.I., G.M.) assessed the methodological quality of the included studies independently and in duplicate. Disagreements over the assessment were resolved by a third author (A.C.). The Methodological Index for non-Randomized Studies (MINORS) [12] tool was used for the qualitative assessment, due to its ability to evaluate the methodological quality of single-arm studies. The items were scored as 0 (not reported), 1 (reported but inadequate) or 2 (reported and adequate). The global ideal score was 16 for non-comparative studies and 24 for comparative studies.

### 2.2. Quantitative Analysis

A meta-analysis was performed in case of two or more included studies reporting data on the outcomes of interest. For the dichotomous outcomes, the summary estimates were derived from logit transformation of individual study proportions and presented along with the corresponding 95% confidence interval (C.I.), calculated using random effect. For the continuous outcome, the summary estimate was derived from one-arm metric mean and standard deviation (SD) of individual study outcome and presented along with the corresponding 95% confidence interval (C.I.), calculated using random effect. Mean and SD were calculated with appropriate formulae when not available [13]. A sensitivity analysis including articles comparing the occurrence of VAP in patients with COVID-19 to those of patients admitted to ICU without COVID-19 was performed, providing an odds ratio as a measure of risk. A sensitivity analysis including articles comparing the risk of death in COVID-19 patients with VAP to those of patients without VAP was also performed, providing an odds ratio as a measure of risk. A subgroup analysis was performed based on the number of centers (e.g., single or multicenter studies). An I-squared (I^2^) statistical model was used to evaluate heterogeneity among the included studies. All the analyses were performed by A.C. and M.I., using Open Meta-Analyst 8 [14].

## 3. Results

### 3.1. Characteristics of Included Studies and Patients

**Figure 1:**
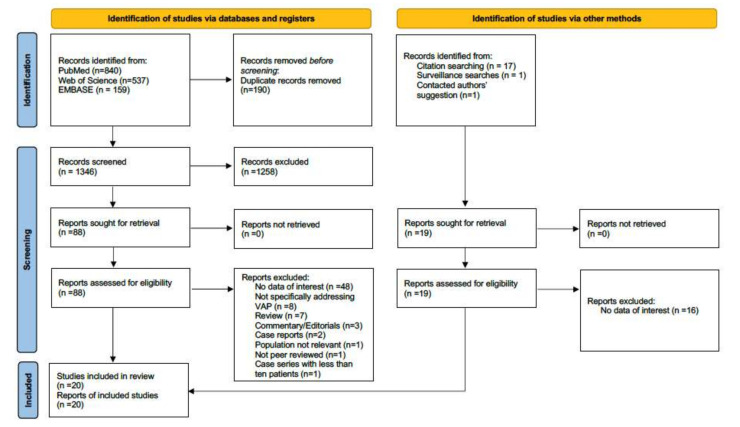
PRISMA 2020 flow diagram for new systematic reviews which included searches of databases, registers and other sources.

A total of 1555 records were retrieved in the comprehensive search. The full search output is available as Supplementary Material S3. After the exclusion of duplicates and not relevant records, twenty studies were included in the systematic review and meta-analysis, for a total of 2611 patients with COVID-19 who developed at least one episode of VAP [9,15,16,17,18,19,20,21,22,23,24,25,26,27,28,29,30,31,32,33]. The process of inclusion and exclusion is detailed in the PRISMA flow diagram, provided in Figure 1.

The characteristics of the included studies are provided in Table 1. Eighteen studies had an observational retrospective design (18/20, 90%), of which five were multicenter [9,17,22,27,32] and thirteen were performed in a single center [15,16,19,20,21,24,25,26,28,29,30,31,32,33]. Two studies had a prospective observational design (2/20, 10%) and were multicenter. All the studies were conducted in the European Union (E.U.), except one in China [27] and one in Russia [24]. The patients evaluated in the included studies had a mean or median age ranging from 49 to 69.5 years, with a percentage of female gender ranging from 18% to 55%. All the studies were single-arm studies, except five also including patients admitted to ICU without COVID-19 as a comparison group [17,19,21,22,28]. The median duration of mechanical ventilation prior to VAP in the included patients ranged from 7 to 13 days. Details on isolated microorganisms are provided in Table 2. The detailed qualitative assessment with individual domain and overall MINORS score per study is provided as Tables S3 and S4, Supplementary Material S2. Only three of the studies reported protocol registration and only one reported information on sample calculation. These two were the most frequently downgraded domains at the qualitative assessment of the included studies.

### 3.2. Outcomes

**Figure 2:**
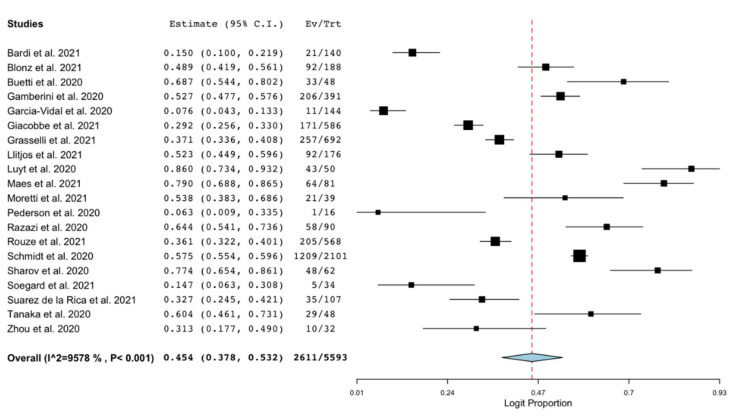
Forest plot with the result of single-arm meta-analysis for the occurrence of VAP in patients with COVID-19. C.I., confidence interval; Ev, events; Trt, total.

All the included studies reported the occurrence of VAP in cohorts of patients with COVID-19 and admitted to ICU. The pooled estimated occurrence of VAP was 45.4% (95% C.I. 37.8–53.2%; 2611/5593 patients; I^2^ = 96%; Figure 2). Cumulative estimates of occurrence of VAP sorted by the sample size of the included studies are provided as Figure S1 in Supplementary Material S2.

**Figure 3:**
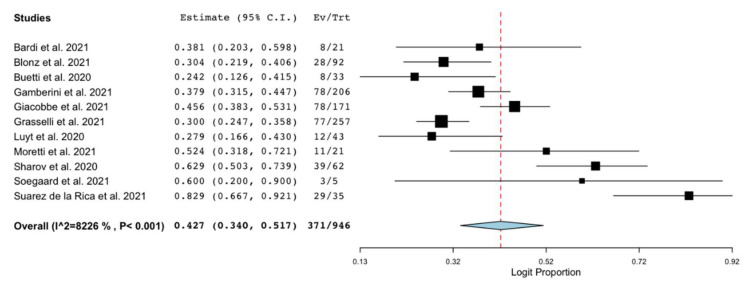
Forest plot with the result of single-arm meta-analysis for mortality of patients with COVID-19 and VAP. C.I., confidence interval; Ev, events; Trt, total.

Eleven of the included studies reported data on mortality in patients with COVID-19 and VAP [9,15,16,20,21,23,24,25,30,31,32]. The pooled estimated occurrence of mortality (at the longest available follow-up) was 42.7% (95% C.I. 34–51.7%; 371/946 patients; I^2^ = 82%; Figure 3). Cumulative estimates of mortality in patients with COVID-19 and VAP sorted by the sample size of the included studies are provided as Figure S2 in Supplementary Material S2.

Seven of the included studies reported data on ICU LOS in patients with COVID-19 and VAP [16,21,23,24,25,31,32], with an estimated summary mean of 28.58 days (95% C.I. 21.4–35.8; I^2^ = 98%; Figure S3, Supplementary Material S2).

### 3.3. Sensitivity and Subgroup Analyses

**Figure 4:**
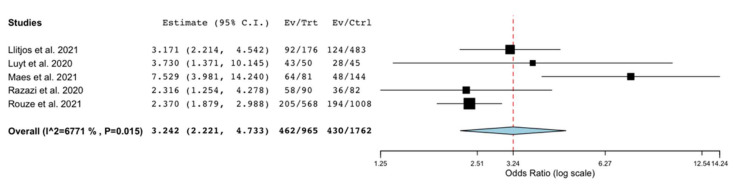
Forest plot with the results of the sensitivity analysis on the occurrence of ventilator-associated pneumonia in patients with COVID-19 compared to patients without COVID-19. C.I., confidence interval; Ctrl, controls; Ev, events; Trt, total.

We conducted a sensitivity analysis only considering the studies reporting data on the occurrence of VAP in patients with COVID-19 and comparison groups of patients admitted to ICU without COVID-19 (Table 1). In this analysis, conducted on the unadjusted data provided by five studies with comparison groups [17,19,21,22,28], we observed a significantly higher risk of VAP in patients with COVID-19, compared to patients without COVID-19 (OR 3.24; 95% C.I. 2.2–4.7; P = 0.015; I^2^ = 67.7%; Figure 4). We also performed a sensitivity analysis including studies reporting data on mortality in patients with COVID-19 and VAP in comparison to the same patients’ cohort who did not develop VAP [15,16,20,21,23,25,30,31]. This unadjusted analysis found no significantly different risk of death in COVID-19 patients with VAP versus non VAP (OR 1.16; 95% C.I. 0.75–1.8; P = 0.007; I^2^ = 62%; Figure S4, Supplementary Material S2).

A subgroup analysis was conducted for the outcome occurrence of VAP based on the number of centers. The pooled estimate of the occurrence of VAP was 42.5% (95% C.I. 32.8–52.7%; 2150/4546 patients; I^2^ = 97%; Figure S5, Supplementary Material S2) when considering only multicenter studies [9,17,18,22,23,27], and 46.1% (95% C.I. 30.8–62.1%; 461/1047 patients; I^2^ = 95%; Figure S6, Supplementary Material S2) when considering only single center studies [15,16,19,20,21,24,25,26,28,29,30,31].

## 4. Discussion

The main finding of this systematic review and meta-analysis was that nearly one patient with COVID-19 out of two may develop VAP during ICU stay. Previous reports showed that VAP occurs in up to 23–40% of patients admitted to ICU [2,34], with variability usually attributable to differences regarding the clinical setting (e.g., countries, staffing), the population of patients (e.g., the reasons for admission, the severity of underlying disease) [35] or the criteria used to define VAP [7]. A higher occurrence in COVID-19 patients in comparison with patients without COVID-19 was also observed in our sensitivity analysis (OR 3.24; 95% C.I. 2.2–4.7; P=0.015), although performed on unadjusted data. We can speculate that the high occurrence of VAP in patients with COVID-19 may be explained by several factors, both disease and not disease related. Patients with COVID-19 admitted to ICU are generally severely hypoxemic, with both parenchymal and microvascular lung damage [36]. Thus, they are reasonably at a high risk of developing VAP, especially if compared with cohorts with a low proportion of patients with ARDS [28] or large mixed cohorts of patients admitted to ICU [37]. The severity of lung damage in patients with COVID-19 pneumonia may lead to an increase in the rate of infections. Furthermore, patients with COVID-19 frequently needed prolonged mechanical ventilation, prone positioning [1] and received immunomodulant therapies, that may have increased their risk of developing VAP [9]. On the other hand, the ICUs may have been overcrowded in the period of peak of COVID-19 pandemic, with a possibly inadequate staffing (e.g., nurse to patient ratio) and more episodes of cross-contaminations. Healthcare workers may have also encountered difficulties related to the wearing of personal protective equipment in the setting of COVID-19-dedicated ICUs [38]. When considered together, these issues may have reduced the adherence to infection control standard and VAP prevention bundles [16]. Although in most centers broad-spectrum antibiotic therapy is administered concomitantly with the rest of the treatment received by these patients, the occurrence of VAP should be specifically considered by the clinicians, for a timely diagnosis and a targeted therapy, especially in the case of atypical presentation of illness.

The evaluation of mortality as the outcome of patients with VAP has been widely discussed as controversial, mainly due to the difference between crude and attributable mortality, relevant to distinguish the increase in risk attributable to a complication (e.g., VAP) of a primary condition (e.g., acute respiratory failure and mechanical ventilation in COVID-19) [39]. Our analysis estimated a pooled occurrence of death in patients with COVID-19 and VAP of 42.7%. These data may be relevant on their own, but no information can be derived on the impact of VAP on mortality from our data. None of the studies reported attributable mortality due to VAP. We explored this issue in a sensitivity analysis showing no significantly different risk of death in COVID-19 patients with VAP versus non VAP, although the estimate was imprecise.

Our study has strengths, such as the comprehensive search and the methodology and reporting according to PRISMA 2020 [11]. Indeed, we were able to retrieve data from corresponding authors of the included studies that were not primarily reported in the published manuscripts. This increased the comprehensiveness and internal validity of the data collection and analysis.

Nevertheless, our analysis has several limitations. First, the overall sample size used to estimate the occurrence of VAP was modest (5593 patients, of whom 2611 developed VAP) if considering the large amount of mechanically ventilated patients with COVID-19 worldwide. Second, no studies were conducted in the United States (U.S.), thus limiting the external validity of our findings, especially considering that the study event definition may vary from E.U. to U.S.-based centers. Our analysis may also have underestimated the occurrence of VAP, as the diagnosis is based on clinical suspicion, and some clinical features of a VAP are common to the condition of COVID-19 itself. Less frequent use of traditional imaging (X-ray, computerized tomography) may have also hindered the identification of such events as VAP. In addition, our aim was mainly descriptive, with no further analysis conducted on associations with potential risk factors. Moreover, we did not collect data on the adequacy of antimicrobial therapy performed or on any factor associated with the outcomes of interest (e.g., use of protocolized preventive strategies for VAP, study period coincidence to peak of infection, etc.). Our analysis also had a high statistical heterogeneity, probably due to a high variability among the studies in microbiological epidemiology, severity of patients’ clinical condition, observation of VAP prevention bundles and ICU capacity in relationship with peaks of COVID-19 outbreaks. However, a strength of this analysis was to provide a reliable estimate of the occurrence of VAP in patients with COVID-19 and of their mortality.

## 5. Conclusions

Nearly half of patients with COVID-19 admitted to ICU may develop VAP, with a pooled estimate of mortality of 42.7% for COVID-19 patients who developed VAP. Data are needed from the U.S., and further studies should evaluate the attributable risk of death of VAP in patients with COVID-19.

## Figures and Tables

**Table 1:** Characteristics of the included studies. The table shows the characteristics of the included studies, as reported by the authors. Data are reported as proportions, percentages, median [IQR] and mean (± SD).

Authors (Year) [REF]	Design (Country)	COVID-19 ICU Patients *	Non-COVID-19 Comparison Population	Criteria Used for the Definition of VAP
Bardi et al. (2021) [16]	Single center retrospective study (Spain)	140 patients with COVID-19 (RT-PCR) admitted to ICU Age 61 years [IQR 57–67] Female 23% **Patients with VAP**: 21 (15%)	NA	According to Centers for Disease Control and Prevention criteria and the Spanish Society of Infectious Diseases and Clinical Microbiology
Blonz et al. (2021) [15]	Single center retrospective study (France)	188 patients with COVID-19 (RT-PCR) admitted to the ICU, who have been receiving IMV for more than 48 h Age: 64 years (±11) Female 22% **Patients with VAP**: 92 (49%) Duration of MV prior to VAP: 10 days	NA	**Timing**: at least 48 h of IMV **Radiological**: two successive chest radiographs or chest CT scans showing new or progressive lung infiltrates **Clinical**: at least one among (i) body temperature > 38.3 °C with no other cause, (ii) leukocytes < 4000/mm^3^ or >12,000/mm^3^, and at least one among (i) new onset of purulent sputum or change in sputum and (ii) worsening gas exchange **Microbiological**: at least one among (i) positive quantitative culture from minimally contaminated LRT specimen (PN 1), using plugged telescopic catheter with a threshold of 10^3^ CFU/mL or a bronchoalveolar lavage with a threshold of 10^4^ CFU/mL, (ii) positive quantitative culture from possibly contaminated LRT specimen (PN 2) using blind endotracheal aspirate with a threshold of 10^6^ CFU/mL, and (iii) positive growth in culture of pleural fluid (PN 3)
Buetti et al. (2020) [20]	Single center retrospective study (Switzerland)	48 patients with COVID-19 admitted to ICU Age 66 years [IQR 60–71] Female 23% **Patients with VAP**: 33 (69%)	NA	**Radiological**: new or progressive and persistent radiographic infiltrates **Clinical suspicion** **Microbiological**: positive microbiological cultures from lower respiratory tract specimens
Gamberini et al. (2020) [23]	Multicenter prospective observational study 15 ICUs (Italy)	391 patients admitted to ICU with COVID-19 (RT-PCR) Age 66 years [IQR 59–72] Female 23% **Patients with VAP**: 206 (53%)	NA	**Timing**: on mechanical ventilation for >2 calendar days on the date of event, with day of ventilator placement being Day 1 and the ventilator was in place on the date of event or the day before **Radiological**: two or more serial chest imaging test results with at least one among new and persistent or progressive and persistent (i) Infiltrate; (ii) Consolidation; (iii) Cavitation **Clinical**: at least one among (i) fever (>38.0 °C or >100.4 °F), (ii) Leukopenia (≤4000 WBC/mm^3^) or leukocytosis (>12,000 WBC/mm^3^), (iii) for adults >70 years old, altered mental status with no other recognized cause, and at least two among (i) new onset of purulent sputum or change in character of sputum or increased respiratory secretions or increased suctioning requirements, (ii) new onset or worsening cough, or dyspnea, or tachypnea, (iii) rales or bronchial breath sounds or (iv) worsening gas exchange
Garcia-Vidal et al. (2020) [33]	Single center retrospective study (Spain)	144 patients admitted to ICU with COVID-19 (RT-PCR) **Patients with VAP:** 11 (7.6%)	NA	NA
Giacobbe et al. (2021) [9]	Multicenter retrospective study 11 ICUs (Italy)	586 patients admitted to the ICU with COVID-19 (RT-PCR for SARS-CoV-2) who have been receiving invasive mechanical ventilation **Patients with VAP**: 171 (29%) Age: 64 [IQR 57–71] Female 20% **Patients with microbiologically confirmed VAP**: 77 (45%, 92 no specimens analyzed) Duration of MV prior to VAP: 9 days [IQR 5–15]	NA	**Timing**: at least 48 h of IMV **Radiological**: new or changing chest X-ray infiltrate/s **Clinical**: both (i) new onset of body temperature ≥ 38 °C or ≤ 35 °C and/or leukocytosis or leukopenia or immature neutrophils and (ii) new onset of suctioned respiratory secretions and/or need for acute ventilator support system changes to enhance oxygenation **Microbiological confirmation**: (criteria not needed for the diagnosis) positive BALF culture for bacterial respiratory pathogens
Grasselli et al. (2021) [32]	Multicenter retrospective study 8 hospitals (Italy)	692 patients admitted to the ICU with COVID-19 (RT-PCR for SARS-CoV-2) who had been receiving invasive mechanical ventilation **Patients with VAP**: 257 (37%)	NA	At least two among: (i) fever, leukocytosis/leucopenia, purulent secretions, (ii) new/progressive radiographic infiltrate, (iii) worsening oxygenation **Microbiological**: bronchoalveolar lavage ≥ 104 CFU/mL or endotracheal aspirate ≥ 105 CFU/mL
Llitjos et al. (2021) [22]	Multicenter retrospective study 7 ICUs (France)	176 patients with COVID-19 (RT-PCR) admitted to the ICU who have been receiving invasive mechanical ventilation for at least 48 h Age 63 years [IQR 55–73] Female 24% **Patients with VAP**: 92 (52%) Duration of MV prior to VAP: 9 days [IQR 6–14]	- 435 patients with bacterial CAP who have been receiving invasive mechanical ventilation Age 66 years [IQR 56–79] Female 32% **Patients with VAP**: 113 (26%) Duration of MV prior to VAP: 9 days [IQR 6–12]; - 48 patients with viral CAP who have been receiving invasive mechanical ventilation Age 72 years [IQR 42–75] Female 48% **Patients with VAP**: 11 (23%) Duration of MV prior to VAP: 7 days [IQR 6.5–14]	ICU-acquired pneumonia **Timing**: at least 48 h of IMV **Clinical**: diagnosis was based on Clinical Pulmonary Infectious Score >6 **Microbiological confirmation**: (criteria not needed for the diagnosis) direct Gram staining and semi-quantitative culture. An independent physician retrospectively assessed the diagnostic accuracy of all episodes of ICU-acquired pneumonia
Luyt et al. (2020) [21]	Single center retrospective study (France)	50 patients with COVID-19 associated ARDS admitted to the ICU and requiring ECMO Age 48 years [IQR 42–56] Female 28% **Patients with VAP**: 43 (86%) Duration of MV prior to VAP: 10 days [IQR 8–16]	45 patients with severe influenza-associated ARDS requiring ECMO Age 58 years [IQR 48–64] Female 38% **Patients with VAP**: 28 (62%) Duration of MV before VAP: 14 (8–19)	**Timing**: at least 48 h of IMV **Radiological**: new and persistent pulmonary infiltrate on chest radiograph (not needed for patients with ARDS) **Clinical**: at least two criteria among (i) temperature ≥ 38 °C (ii) white blood cell count ≥ 10 Giga/L (iii) purulent tracheal secretions iv) increased minute ventilation (v) arterial oxygenation decline requiring modifications of the ventilator settings and/or (vi) need for increased vasopressor infusion Microbiological: significant quantitative growth (≥10^4^ colony-forming units/mL) of distal BALF samples
Maes et al. (2021) [28]	Single center retrospective study (UK)	81 patients with COVID-19 admitted to the ICU, who have been receiving invasive mechanical ventilation for more than 48 h Age 62 years [IQR 50–70] Female 31% **Patients with VAP**: 64 (79%) Duration of MV prior to VAP: 7 days [IQR 5–12] **Patients with microbiologically confirmed VAP**: 39 (48%)	144 patients admitted to ICU without COVID-19 who have been receiving invasive mechanical ventilation for more than 48 h Age 62 years [IQR 49–72] Female 40% **Patients with VAP**: 48 (34%) Duration of MV prior to VAP: 6 days [IQR 4–9] **Patients with microbiologically confirmed VAP**: 19 (13%)	**Timing**: at least 48 h of IMV **Radiological**: new or worsening infiltrates on Chest X-ray or CT thorax **Clinical**: at least one among (i) fever > 38 °C with no other cause, (ii) leukopenia (<4000 WBC/mm^3^) or leukocytosis (≥12,000 WBC/mm^3^), (iii) new onset of suctioned respiratory secretions or suggestive auscultation or worsening gas exchanges **Microbiological confirmation**: (criteria not needed for the diagnosis) at least one among (i) positive quantitative culture from minimally contaminated LRT specimen (PN 1) or broncho-alveolar lavage with a threshold of ≥ 10^4^ CFU/mL or detection by TaqMan array with Ct ≤ 32 and (ii) positive quantitative culture from possibly contaminated LRT specimen (PN 2) or quantitative culture of LRT specimen with a threshold of 10^5^ CFU/mL
Moretti et al. (2021) [31]	Single center retrospective study (Belgium)	39 patients with COVID-19 (RT-PCR) admitted to the ICU, who have been receiving invasive mechanical ventilation Age 62 [IQR 55–72] Female 28% **Patients with VAP**: 21 (69%) Duration of MV prior to VAP: 13 days [IQR 7–21]	NA	National Healthcare Safety Network (NHSN) 2017. **Clinical**: (required for iVAC, possible and probable VAP) Oxygenation problem occurred with hypothermia (temperature < 36 °C) or fever (temperature > 38 °C) or leukocytosis (>12,000 white blood cells/ mm^3^) or leukocytopenia (<4000 white blood cells/mm^3^) **Microbiological**: (i) (required for possible VAP) qualitative pulmonary infection (endotracheal aspiration or BAL showing on gram stain >25 neutrophils and <10 epithelial cells per low power field) or (ii) (required for probable VAP) a quantitative pulmonary infection (endotracheal aspiration or BAL growing, respectively, >10^5^ CFU/mL and >10^4^ CFU/mL)
Pedersen et al. (2020) [29]	Single center retrospective study (Denmark)	16 patients with COVID-19 admitted to ICU 69.5 years (range: 56–84 years) Female 25% **Patients with VAP**: 1 (6%)	NA	NA
Razazi et al. (2020) [19]	Single center retrospective study (France)	90 patients with COVID-19 (RT-PCR) associated ARDS admitted to ICU, who required mechanical ventilation for more than 48 h Age 59 [IQR 53–69] Female 18% **Patients with VAP**: 58 (64%) Duration of MV prior to VAP: 8 days [IQR 5–12]	82 patients admitted to ICU with non-COVID-19 associated ARDS (50 with severe influenza) Age 63 [IQR 57–71] Female 34% **Patients with VAP**: 36 (44%) Duration of MV prior to VAP: 7 days [IQR 5–9]	**Timing**: at least 48 h of IMV **Radiological**: new or worsening infiltrates on chest X-ray **Clinical**: systemic signs of infection (new-onset fever, leukocytosis or leucopenia, increased need for vasopressors to maintain blood pressure), purulent secretions, and impaired oxygenation **Microbiological**: quantitative cultures of lower respiratory tract secretions sampled before administering new antibiotics using a blinded protected telescope catheter or bronchoscopy (10^3^ and 10^4^ colony forming units/ mL, respectively)
Rouzè et al. (2021) [17]	Multicenter retrospective study 36 ICUs (28 in France, 3 in Spain, 3 in Greece, 1 in Portugal, 1 in Ireland)	568 patients with COVID-19, admitted to ICU, who have been receiving invasive mechanical ventilation for more than 48 h Age 64 years [IQR 55–71] Female 41% **Patients with VAP**: 205 (36%)	- 482 patients admitted to ICU with influenza pneumonia who required mechanical ventilation for more than 48 h Age 62 years [IQR 53–71] Female 49% **Patients with VAP**: 107 (22%) - 526 patients admitted to ICU with no viral infection who required mechanical ventilation for more than 48 h Age 65 years [IQR 55–74] Female 45% **Patients with VAP**: 87 (16%)	**Timing**: at least 48 h of IMV **Clinical**: at least two among (i) body temperature of more than 38.5 °C or less than 36.5 °C, (ii) leucocyte count greater than 12,000 cells per μL or less than 4000 cells per μL, and (iii) purulent tracheal secretions **Radiological**: new or progressive infiltrates on chest X-ray **Microbiological**: isolation in the endotracheal aspirate of at least 10^5^ CFU per mL, or in bronchoalveolar lavage of at least 10^4^ CFU per mL
Schmidt et al. (2020) [18]	Multicenter prospective cohort study 149 ICUs (France, Switzerland, Belgium)	2101 patients with COVID-19 (RT-PCR) admitted to ICU and intubated on day 1 **Patients with VAP**: 1209/2101 (58%)	NA	**Clinical suspicion** **Microbiological**: quantitative distal bronchoalveolar lavage cultures growing ≥ 10^4^ CFU/mL, blind protected specimen brush distal growing ≥ 10^3^ CFU/mL, or endotracheal aspirates growing ≥ 10^6^ CFU/mL
Sharov et al. (2020) [24]	Single center retrospective study (Russia)	62 patients with COVID-19 (molecular biological techniques) and mechanically ventilated Age 54.2 years ± 15.3 Female 55% **Patients with VAP**: 48 (77%) Duration of MV prior to VAP: 9.1 days ± 5.6	NA	**Timing**: at least after 3.5 days of IMV **Radiological**: AI-based algorithm of CT images **Microbiological**: biochemical methods
Søgaard et al. (2021) [25]	Single center retrospective study (Switzerland)	34 patients with COVID-19 (RT-PCR) admitted to ICU who required mechanical ventilation Age 65 years [IQR 55–72] Female 24% **Patients with VAP**: 5 (15%) **Patients with microbiologically confirmed VAP:** 4 (12%)	NA	**Timing**: at least 48 h of IMV **Radiological**: consolidations consistent with bacterial pneumonia **Clinical**: indicators of worsening oxygenation, new fever and purulent respiratory secretions **Microbiological confirmation**: (criteria not needed for the diagnosis) positive culture for a respiratory pathogen
Suarez de la Rica et al. (2021) [30]	Single center retrospective study (Spain)	107 patients with COVID-19 (RT-PCR) admitted to ICU and mechanically ventilated Age 62.2 years ± 10.6 Female 29% **Patients with VAP**: 35 (32.7%)	NA	According to Centers for Disease Control (CDC) criteria **Clinical suspicion** **Radiological criteria** **Microbiological**: at least 1 bacterial species by conventional culture, with a threshold of ≥10^5^ colony forming units in endotracheal aspirates.
Tanaka et al. (2020) [26]	Single center retrospective study (France)	48 patients with COVID-19 ARDS admitted to ICU Age 57 [IQR 46-64] Female 35% **Patients with VAP**: 29 (60.4%)	NA	According to the Infectious Diseases Society of America and the American Thoracic Society guidelines
Zhou et al. (2020) [27]	Multicenter retrospective study (China)	32 patients with COVID-19 (according to WHO interim guidance) requiring invasive mechanical ventilation **Patients with VAP**: 10 (31%)	NA	According to ATS guidelines for treatment of hospital-acquired and ventilator-associated pneumonia

* For the studies conducted in centers where microbiological documentation was not mandatory to establish VAP diagnosis, we reported the number of patients with VAP and the number of patients with microbiologically confirmed VAP. ARDS, acute respiratory distress syndrome; ATS, American Thoracic Society; BAL, bronchoalveolar lavage; CAP, community-acquired pneumonia; CFU, colony forming unit; ICU, intensive care unit; IMV, invasive mechanical ventilation; LRT, low respiratory tract; RT-PCR, reverse transcriptase-polymerase chain reaction; VAP, ventilator-associated pneumonia; WHO, World Health Organization.

**Table 2:** Main microorganisms isolated in patients with COVID-19 and VAP. The table shows the number of isolates containing *E. faecium*, *S. aureus*, *K. pneumonia*, *A. baumannii, P. aeruginosa*, *Enterobater* spp., and *E. coli* in patients with COVID-19 and VAP per each included study, if available. The microorganisms are sorted by families. Data are shown as numbers and percentages. All the percentages are calculated on the total number of isolates, when available. When data on these individual microorganisms were not available, we reported them as grouped by the authors.

Authors (Year) [REF]	Microorganisms *n* Isolates (%)	Antimicrobial Resistance *n* Isolates (%)
Bardi et al. (2021) [16]	Enterobacteriaceae *E. cloacae,* 1 (5%) Gram-positive *S. aureus, 5 (24%)* Non-fermenting Gram-negative *A. baumannii, 1 (5%)* *P. aeruginosa, 8 (38%)*	MDR bacteria, 10 (48%) MRSA, 5 (24%)
Blonz et al. (2021) [15]	Enterobacteriaceae, 102 (49%) *E. cloacae,* 10 *E. coli,* 26 *K. pneumoniae,* 16 Gram-positive *E. faecium*, 1 (0.5%) *S. aureus,* 28 (14%) Non-fermenting Gram-negative *A. baumannii,* 4 (2%) *P. aeruginosa,* 31 (15%)	MRSA, 3 (1.4%)
Garcia-Vidal et al. (2020) [33]	Enterobacteriaceae *K. pneumoniae* 1 Non-fermenting Gram-negative *P. aeruginosa* 3 Gram-positive *S. aureus* 4	NA
Giacobbe et al. (2021) [9]	Enterobacteriaceae *E. coli,* 2 (3%) *E. aerogenes,* 7 (9%) *K. pneumoniae,* 15 (19%) Gram-positive *S. aureus,* 18 (23%) Non-fermenting Gram-negative *Acinetobacter* spp., 9 (12%) *P. aeruginosa,* 27 (35%)	MRSA, 8 (10%) Carbapenem-resistant Gram-negative bacteria, 25 (32%)
Grasselli et al. (2021) [32]	Gram-negative 249 (64%) *P. aeruginosa*, 85 Enterobacterales (other) 53 *Klebsiella* spp., 43 *E. coli*, 31 *A. baumanii*, 6 Gram-positive 140 (36%) *Staphylococcus aureus*, 110 *Enterococcus* spp., 21	NA
Llitjos et al. (2021) [22]	Enterobacteriaceae, 50 (NA) Non-fermenting Gram-negative, 20 (NA) Gram-positive cocci, 28 (NA) Polymicrobial, 24 (NA)	NA
Luyt et al. * (2020) [21]	Enterobacteriaceae, 30 (70%) *E. cloacae*, 3 Gram-positive *S. aureus*, 3 (7%) Non-fermenting Gram-negative, 18 (42%) *P. aeruginosa,* 16	Inducible AmpC Enterobacteriaceae, 17 (40%) ESBL-PE, 2 (5%) MRSA, 2 (5%)
Maes et al. (2021) [28]	Enterobacteriaceae *E. coli*, 2 (NA) *K. pneumoniae,* 3 (NA) Gram-positive *S. aureus,* 2 (NA) Non-fermenting Gram-negative *P. aeruginosa,* 3 (NA)	NA
Moretti et al. (2021) [31]	Enterobacteriaceae *Enterobacter* spp., NA (11%) *K. pneumoniae*, NA (26%) Gram-positive *S. aureus*, NA (7%) Non-fermenting Gram-negative *P. aeruginosa,* NA (18%)	MDR, 67% XDR, 1 (5%)
Pedersen et al. [29]	Enterobacteriaceae *E. cloacae* 1 (100%)	
Razazi et al. (2020) * [19]	Enterobacteriaceae, 42 (72%) *Enterobacter* spp., 23 *E. coli,* 10 *K. pneumoniae,* 4 Non-fermenting Gram-negative, 24 (41%) *Acinetobacter* spp., 1 *Pseudomonas* spp., 16 Gram-positive, 4 (3%) *S. aureus* 2	MDR VAP, 21 (23%) ESBL-PE, 18 (20%) CRE, 3 (3%) MRSA plus ESBL-PE, 1 (1%)
Rouzè et al. (2021) ^#^ [17]	Gram-negative bacilli, 240 (83.6%) Enterobacteriaceae *Enterobacter* spp., 54 *E. coli*, 24 *Klebsiella* spp., 33 Non-fermenting Gram-negative *A. baumannii,* 2 *P. aeruginosa,* 64 Gram-positive cocci, 56 (19.5%) *Enterococcus* spp., 9 *S. aureus*, 35	Multidrug-resistant isolates, 67 (23%) MRSA, 8 (3%)
Sharov et al. (2020) [24] (data from the private scientific correspondence with Dr. K.S. Sharov and Dr. A.S. Gorenintseva from the Russian Academy of Sciences)	Enterobacteriaceae *K. pneumoniae*, 5 (8%) Gram-positive *S. aureus*, 8 (13%)	NA
Søgaard et al. (2021) [25]	Enterobacteriaceae *E. coli,* 1 (20%) Non-fermenting Gram-negative *A. baumannii,* 1 (20%)	MDR, 1 (20%)
Suarez de la Rica et al. (2021) [30]	Enterobacteriaceae *Enterobacter* spp., 1 (2.8%) *E. coli*, 4 (11.4%) *Klebsiella* spp., 9 (26%) Non-fermenting Gram-negative *P. aeruginosa,* 11 (31%) Gram-positive *S. aureus*, 8 (23%)	NA

* Data regards only the first VAP episode. ^#^ Data provided on the cohort of LRTI, including VAP. CRE, carbapeneme-resistant enterobacteriacae; ESBL, extended-spectrum beta-lactamase; ESBL-PE, extended-spectrum beta-lactamase producing enterobacteriacae; MDR, multidrug resistant; MRSA, methicillin-resistant S. aureus; NA, not available; VAP, ventilator-associated pneumonia; XDR, extensively drug resistant.

## Data Availability

The data presented in this study are available in Supplementary Materials. The unpublished data retrieved by private correspondence with the authors and used in this study analysis are available on request from the corresponding author.

## Supplementary Materials

The following are available online: https://www.mdpi.com/article/10.3390/antibiotics10050545/s1, Supplementary Material S1: Search strategy; Supplementary Material S2: Figure S1: Forest plots with the results of cumulative single-arm meta-analysis for the occurrence of ventilator-associated pneumonia in patients with COVID-19; Figure S2: Forest plots with the results of cumulative single-arm meta-analysis for the mortality of patients with COVID-19 and ventilator-associated pneumonia; Figure S3: Forest plot with the results of single-arm meta-analysis for intensive care unit length of stay of patients with COVID-19 and ventilator-associated pneumonia; Figure S4: Forest plot with the results of the sensitivity analysis on the mortality of patients with COVID-19 and ventilator-associated pneumonia compared to patients with COVID-19 and admitted to ICU who did not develop ventilator-associated pneumonia; Figure S5: Forest plot with the results of the subgroup analysis conducted on the occurrence of ventilator-associated pneumonia in patients with COVID-19 in multicenter studies; Figure S6: Forest plot with the results of the subgroup analysis conducted on the occurrence of ventilator-associated pneumonia in patients with COVID-19 in single center studies; Table S1: PRISMA Main Checklist; Table S2: PRISMA Abstract Checklist; Table S3: Quality assessment of studies according to MINORS score for non-comparative studies; Table S4: Quality assessment of studies according to MINORS additional score for comparative studies; Supplementary Material S3: Full search output.
