# Supplementary material for: Ventilator-Associated Pneumonia in Patients with COVID-19: A Systematic Review and Meta-Analysis
Source: Antibiotics (Basel). 2021 May 7;10(5):545. doi: 10.3390/antibiotics10050545 (PMC8150614; doi:10.3390/antibiotics10050545)
Supplement: Supplementary file 1 [file antibiotics-10-00545-s001.zip › Suppl. 1.pdf]

Ventilator-Associated Pneumonia in patients with COVID-19:
A systematic review and meta-analysis

Mariachiara Ippolito[1], Giovanni Misseri[2], Giulia Catalisano[1], Claudia Marino[1], Giulia Ingoglia[1], Marta Alessi[1], Elisa Consiglio[1], Cesare Gregoretti[1,2], Antonino Giarratano[1,3], Andrea Cortegiani[1,3,*]

1 Department of Surgical, Oncological and Oral Science (Di.Chir.On.S.). University of Palermo, Italy;
MI mariachiara.ippolito@community.unipa.it; GC, giuliacatalisano@gmail.com; CM, dott.ssacmarino@gmail.com; GI, ingogiulia@gmail.com; MA, martalessi@hotmail.it; EC, elisa.consiglio0306@gmail.com; CG, cesare.gregoretti@unipa.it; AG, antonino.giarratano@unipa.it; AC, andrea.cortegiani@unipa.it

2 Fondazione "Giglio", Cefalù, Italy; GM, giovannimisseri1987@gmail.com; CG, cesare.gregoretti@unipa.it;

3 Department of Anaesthesia, Intensive Care and Emergency, Policlinico Paolo Giaccone, Palermo, Italy; AG, antonino.giarratano@unipa.it; AC, andrea.cortegiani@unipa.it

* Correspondence: andrea.cortegiani@unipa.it; Tel.: +390916552730 (University of Palermo. Department of Anaesthesia, Intensive Care and Emergency, Policlinico Paolo Giaccone, Palermo, Italy. Via del Vespro 129, 90127 Palermo, Italy.)

Search strategy

PUBMED

Search: **('ventilator associated pneumonia' OR 'VAP' OR 'healthcare-associated pneumonia') AND ('coronavirus disease 2019' OR 'sars cov 2' OR 'covid')**

("pneumonia, ventilator associated"[MeSH Terms] OR ("pneumonia"[All Fields] AND "ventilator associated"[All Fields]) OR "ventilator-associated pneumonia"[All Fields] OR ("ventilator"[All Fields] AND "associated"[All Fields] AND "pneumonia"[All Fields]) OR "ventilator associated pneumonia"[All Fields] OR "vap" [All Fields] OR ("healthcare associated pneumonia"[MeSH Terms] OR ("healthcare associated"[All Fields] AND "pneumonia"[All Fields]) OR "healthcare associated pneumonia"[All Fields] OR ("healthcare"[All Fields] AND "associated"[All Fields] AND "pneumonia"[All Fields]) OR "healthcare associated pneumonia"[All Fields])) AND ("covid 19"[MeSH Terms] OR "covid 19"[All Fields] OR "coronavirus disease 2019"[All Fields] OR ("sars cov 2"[MeSH Terms] OR "sars cov 2"[All Fields] OR "sars cov 2"[All Fields]) OR ("sars cov 2"[MeSH Terms] OR "sars cov 2"[All Fields] OR "covid"[All Fields] OR "covid 19"[MeSH Terms] OR "covid 19"[All Fields]))

Translations

'ventilator associated pneumonia':"pneumonia, ventilator-associated"[MeSH Terms] OR ("pneumonia"[All Fields] AND "ventilator-associated"[All Fields]) OR "ventilator-associated pneumonia"[All Fields] OR ("ventilator"[All Fields] AND "associated"[All Fields] AND "pneumonia"[All Fields]) OR "ventilator associated pneumonia"[All Fields]

'healthcare-associated pneumonia':"healthcare-associated pneumonia"[MeSH Terms] OR ("healthcare-associated"[All Fields] AND "pneumonia"[All Fields]) OR "healthcare-associated pneumonia"[All Fields] OR ("healthcare"[All Fields] AND "associated"[All Fields] AND "pneumonia"[All Fields]) OR "healthcare associated pneumonia"[All Fields]

'coronavirus disease 2019': "covid-19"[MeSH Terms] OR "covid-19"[All Fields] OR "coronavirus disease 2019"[All Fields]

'sars cov 2': "sars-cov-2"[MeSH Terms] OR "sars-cov-2"[All Fields] OR "sars cov 2"[All Fields]

'covid': "sars-cov-2"[MeSH Terms] OR "sars-cov-2"[All Fields] OR "covid"[All Fields] OR "covid-19"[MeSH Terms] OR "covid-19"[All Fields]

EMBASE

('ventilator associated pneumonia'/exp OR 'health care associated pneumonia'/exp OR 'vap' OR 'ventilator associated pneumonia' OR 'healthcare associated pneumonia') AND ('coronavirus disease 2019'/exp OR 'sars cov 2/exp' OR 'covid19'/exp OR 'covid' OR 'sars-cov-2') AND ([embase]/lim OR [medline]/lim OR [pubmed-not-medline]/lim)

WOS

(ALL=ventilator-associated pneumonia OR ALL= healthcare-associated pneumonia OR ALL= health care associated pneumonia OR ALL= healthcare associated pneumonia OR ALL= vap OR ALL= ventilator associated pneumonia) AND (ALL=coronavirus disease 2019 OR ALL=sars-cov-2 OR ALL= COVID-19 OR ALL=covid19)
