# Supplementary material for: Ventilator-Associated Pneumonia in Patients with COVID-19: A Systematic Review and Meta-Analysis
Source: Antibiotics (Basel). 2021 May 7;10(5):545. doi: 10.3390/antibiotics10050545 (PMC8150614; doi:10.3390/antibiotics10050545)
Supplement: Supplementary file 1 [file antibiotics-10-00545-s001.zip › Supplementary Material 2.pdf]

Supplementary Material to:

Ventilator-Associated Pneumonia in patients with COVID-19: A systematic review and meta-analysis

Mariachiara Ippolito[1], Giovanni Misseri[2], Giulia Catalisano[1], Claudia Marino[1], Giulia Ingoglia[1], Marta Alessi[1], Elisa Consiglio[1], Cesare Gregoretti[1,2], Antonino Giarratano[1,3], Andrea Cortegiani[1,3,*]

[1] Department of Surgical, Oncological and Oral Science (Di.Chir.On.S.). University of Palermo, Italy; MI mariachiara.ippolito@community.unipa.it; GC, giuliacatalisano@gmail.com; CM, dott.ssacmarino@gmail.com; GI, ingogiulia@gmail.com; MA, martalessi@hotmail.it; EC, elisa.consiglio0306@gmail.com; CG, cesare.gregoretti@unipa.it; AG, antonino.giarratano@unipa.it; AC, andrea.cortegiani@unipa.it

[2] Fondazione "Giglio", Cefalù, Italy; GM, giovannimisseri1987@gmail.com; CG, cesare.gregoretti@unipa.it;

[3] Department of Anaesthesia, Intensive Care and Emergency, Policlinico Paolo Giaccone, Palermo, Italy; AG, antonino.giarratano@unipa.it; AC, andrea.cortegiani@unipa.it

* Correspondence: andrea.cortegiani@unipa.it; Tel.: +390916552730 (University of Palermo. Department of Anaesthesia, Intensive Care and Emergency, Policlinico Paolo Giaccone, Palermo, Italy. Via del Vespro 129, 90127 Palermo, Italy.)

Contents

Figure S1. Forest plots with the results of cumulative single arm meta-analysis for the occurrence of ventilator-associated pneumonia in patients with COVID-19

Figure S2. Forest plots with the results of cumulative single arm meta-analysis for the mortality of patients with COVID-19 and ventilator-associated pneumonia

Figure S3. Forest plot with the results of single arm meta-analysis for intensive care unit length of stay of patients with COVID-19 and ventilator-associated pneumonia

Figure S4. Forest plot with the results of the sensitivity analysis on the mortality of patients with COVID-19 and ventilator-associated pneumonia compared to patients with COVID-19 and admitted to ICU who did not develop ventilator-associated pneumonia

Figure S5. Forest plot with the results of the subgroup analysis conducted on the occurrence of ventilator-associated pneumonia in patients with COVID-19 in multicentre studies

Figure S6. Forest plot with the results of the subgroup analysis conducted on the occurrence of ventilator-associated pneumonia in patients with COVID-19 in single centre studies

Table S1. PRISMA Main Checklist

Table S2. PRISMA Abstract Checklist

Table S3. Quality assessment of studies according to MINORS score for non-comparative studies

Table S4. Quality assessment of studies according to MINORS additional score for comparative studies

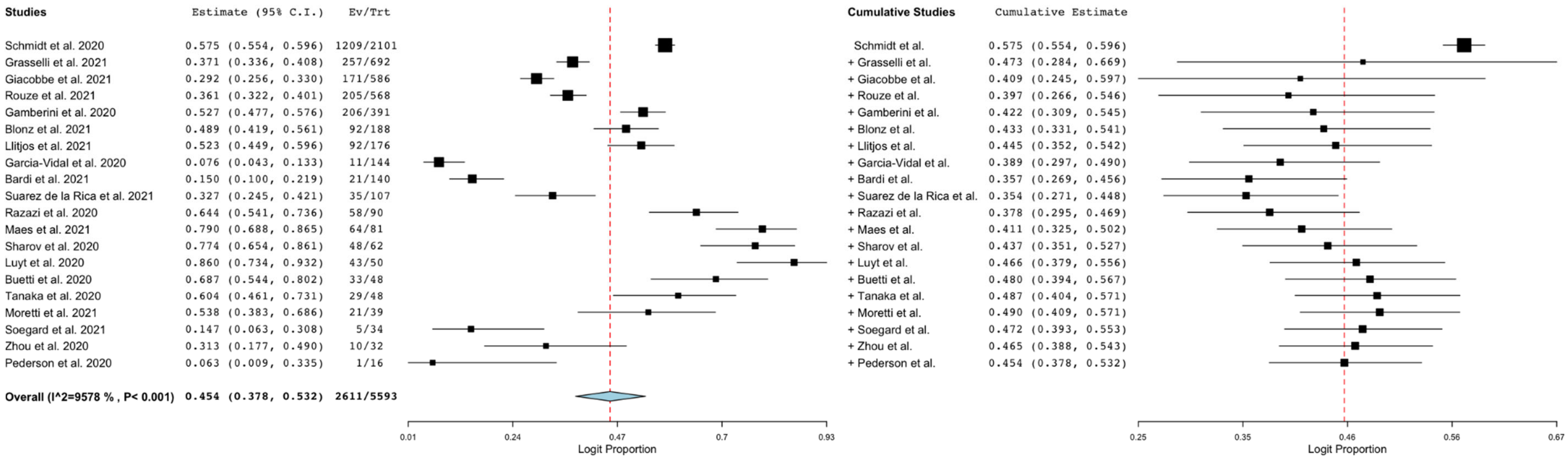

Figure S1. Forest plots with the results of cumulative single arm meta-analysis for the occurrence of ventilator-associated pneumonia in patients with COVID-19

The figure shows the forest plots with results of cumulative single arm meta-analysis for the occurrence of ventilator-associated pneumonia in patients with COVID-19.

Studies are sorted by sample size.

C.I., confidence interval; Ev, events; Trt, total

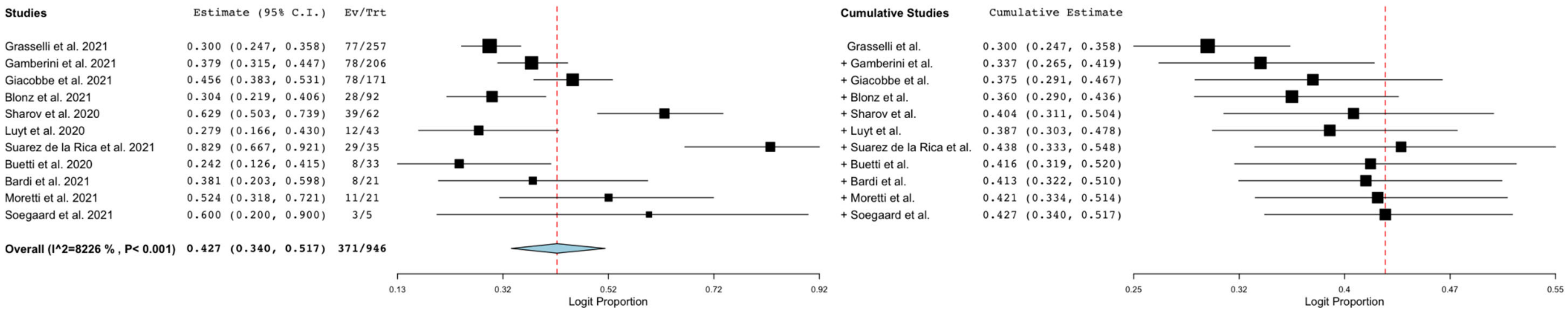

Figure S2. Forest plots with the results of cumulative single arm meta-analysis for the mortality of patients with COVID-19 and ventilator-associated pneumonia

The figure shows the forest plot with the results of cumulative single arm meta-analysis for the mortality of patients with COVID-19 and ventilator-associated pneumonia.

Studies were sorted by sample size.

C.I., confidence interval; Ev, events; Trt, total

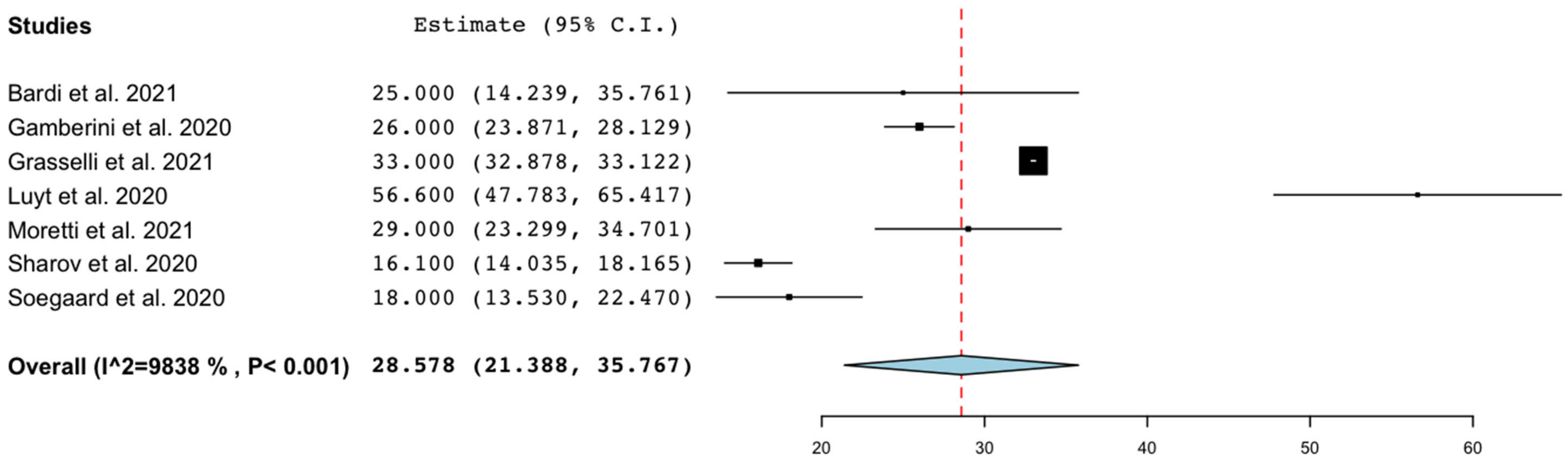

Figure S3. Forest plot with the results of single arm meta-analysis for intensive care unit length of stay of patients with COVID-19 and ventilator-associated pneumonia

The figure shows the forest plot with the results of single arm meta-analysis for intensive care unit length of stay of patients with COVID-19 and ventilator-associated pneumonia.

C.I., confidence interval

Studies	Estimate (95% C.I.)	Ev/Trt	Ev/Ctrl
Bardi et al. 2021	1.088 (0.418, 2.832)	8/21	43/119
Blonz et al. 2021	1.178 (0.626, 2.217)	28/92	26/96
Buetti et al. 2020	0.880 (0.218, 3.547)	8/33	4/15
Gamberini et al. 2021	1.180 (0.780, 1.786)	78/206	63/185
Grasselli et al. 2021	0.685 (0.504, 0.932)	77/257	266/692
Luyt et al. 2020	0.155 (0.026, 0.909)	12/43	5/7
Moretti et al. 2021	2.200 (0.599, 8.084)	11/21	6/18
Soegaard et al. 2021	9.300 (1.230, 70.333)	3/5	5/36
Suarez de la Rica et al. 2021	2.900 (1.067, 7.884)	29/35	45/72
**Overall (I^2=6230 % , P=0.007)**	**1.159 (0.747, 1.796)**	**254/713**	**463/1240**

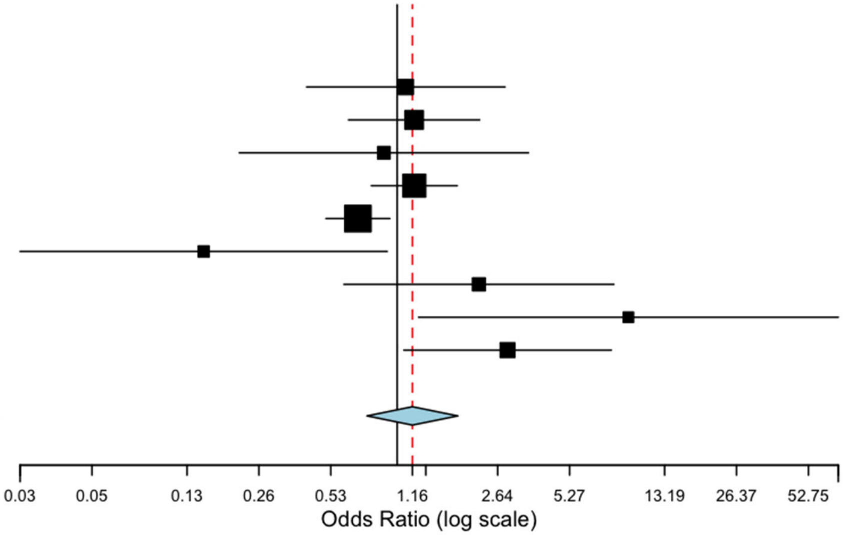

Figure S4. Forest plot with the results of the sensitivity analysis on the mortality of patients with COVID-19 and ventilator-associated pneumonia compared to patients with COVID-19 and admitted to ICU who did not develop ventilator-associated pneumonia

The figure shows the forest plot with the results of the sensitivity analysis on the mortality of patients with COVID-19 and ventilator-associated pneumonia compared to patients with COVID-19 and admitted to ICU who did not develop ventilator-associated pneumonia

C.I., confidence interval; Ctrl, controls; Ev, events; Trt, total

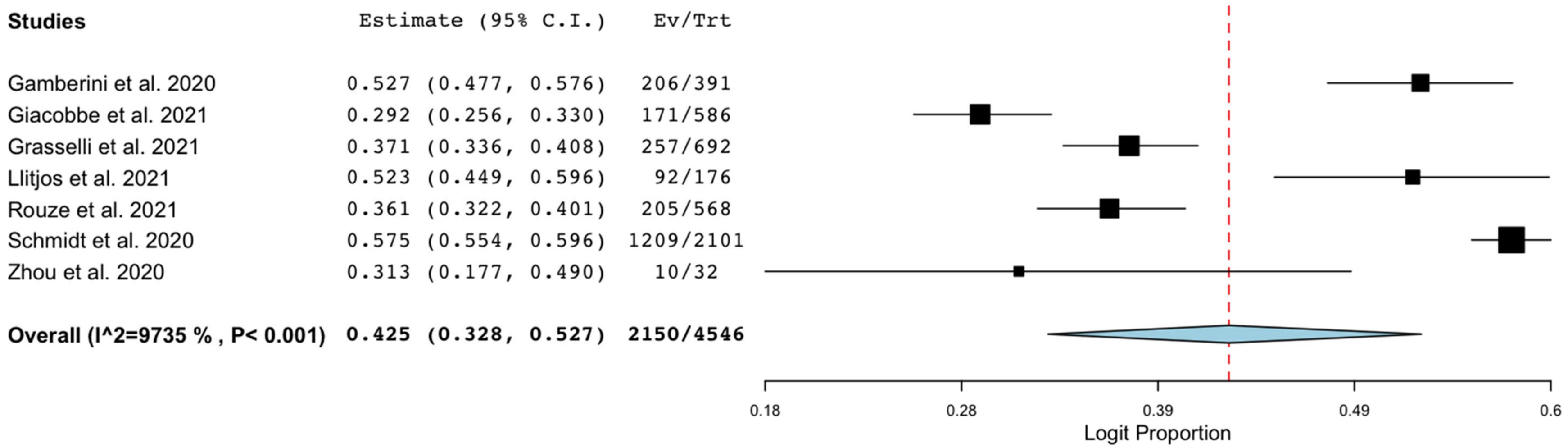

Figure S5. Forest plot with the results of the subgroup analysis conducted on the occurrence of ventilator-associated pneumonia in patients with COVID-19 in multicentre studies

The figure shows the results of the subgroup analysis conducted on the occurrence of ventilator-associated pneumonia in patients with COVID-19 in multicentre studies.

C.I., confidence interval; Ev, events; Trt, total

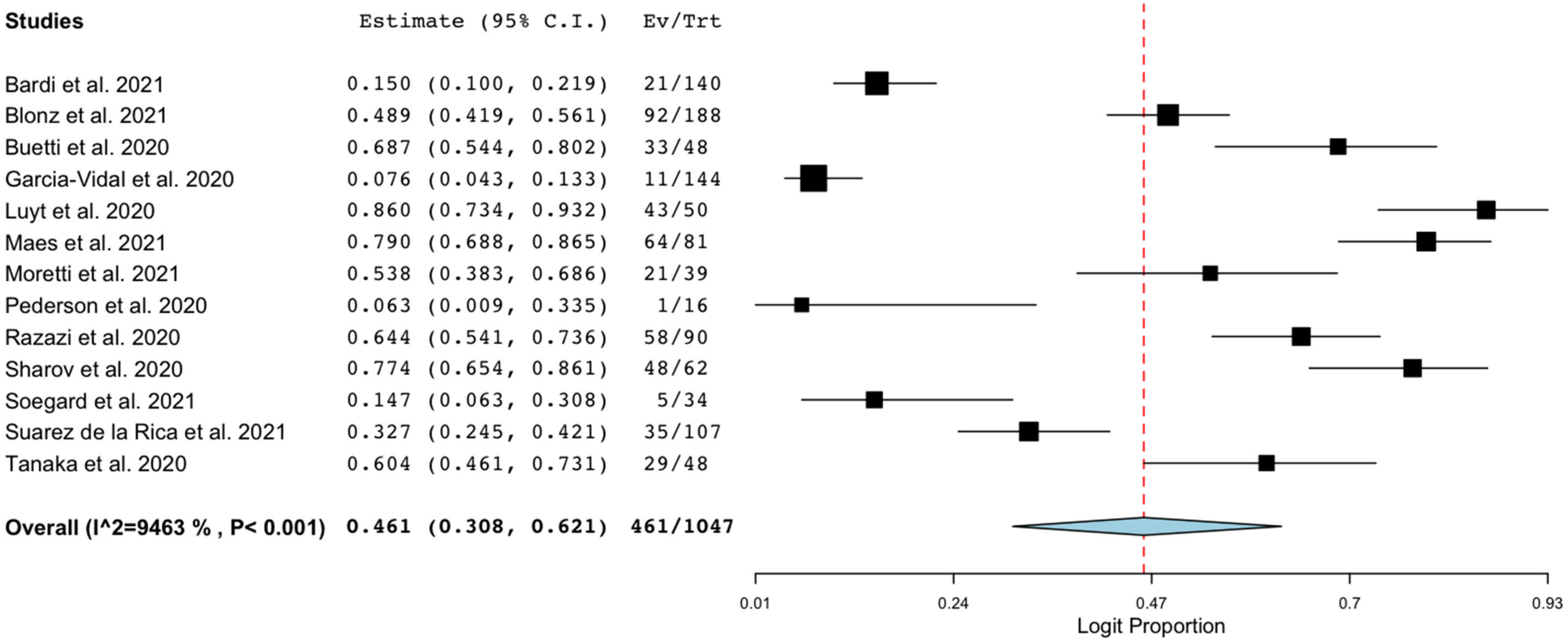

Figure S6. Forest plot with the results of the subgroup analysis conducted on the occurrence of ventilator-associated pneumonia in patients with COVID-19 in single centre studies

The figure shows the results of the subgroup analysis conducted on the occurrence of ventilator-associated pneumonia in patients with COVID-19 in single centre studies.

C.I., confidence interval; Ev, events; Trt, total

Topic	No.	Item	Location where item is reported
**TITLE**			
**Title**	1	Identify the report as a systematic review.	Page 1
**ABSTRACT**			
**Abstract**	2	See the PRISMA 2020 for Abstracts checklist	Table S2
**INTRODUCTION**			
**Rationale**	3	Describe the rationale for the review in the context of existing knowledge.	Line 37-57
**Objectives**	4	Provide an explicit statement of the objective(s) or question(s) the review addresses.	Line 58-63
**METHODS**			
**Eligibility criteria**	5	Specify the inclusion and exclusion criteria for the review and how studies were grouped for the syntheses.	Line 67-74
**Information sources**	6	Specify all databases, registers, websites, organisations, reference lists and other sources searched or consulted to identify studies. Specify the date when each source was last searched or consulted.	Line 67
**Search strategy**	7	Present the full search strategies for all databases, registers and websites, including any filters and limits used.	Supplementary Material 1
**Selection process**	8	Specify the methods used to decide whether a study met the inclusion criteria of the review, including how many reviewers screened each record and each report retrieved, whether they worked independently, and if applicable, details of automation tools used in the process.	Line 69-84
**Data collection process**	9	Specify the methods used to collect data from reports, including how many reviewers collected data from each report, whether they worked independently, any processes for obtaining or confirming data from study investigators, and if applicable, details of automation tools used in the process.	Line 85-93
**Data items**	10a	List and define all outcomes for which data were sought. Specify whether all results that were compatible with each outcome domain in each study were sought (e.g. for all measures, time points, analyses), and if not, the methods used to decide which results to collect.	Line 72-73
	10b	List and define all other variables for which data were sought (e.g. participant and intervention characteristics, funding sources). Describe any assumptions made about any missing or unclear information.	Line 86-89

Topic	No.	Item	Location where item is reported
**Study risk of bias assessment**	11	Specify the methods used to assess risk of bias in the included studies, including details of the tool(s) used, how many reviewers assessed each study and whether they worked independently, and if applicable, details of automation tools used in the process.	Line 94-101
**Effect measures**	12	Specify for each outcome the effect measure(s) (e.g. risk ratio, mean difference) used in the synthesis or presentation of results.	Line 103-126
**Synthesis methods**	13a	Describe the processes used to decide which studies were eligible for each synthesis (e.g. tabulating the study intervention characteristics and comparing against the planned groups for each synthesis (item 5)).	NA
	13b	Describe any methods required to prepare the data for presentation or synthesis, such as handling of missing summary statistics, or data conversions.	Line 103-104
	13c	Describe any methods used to tabulate or visually display results of individual studies and syntheses.	NA
	13d	Describe any methods used to synthesize results and provide a rationale for the choice(s). If meta-analysis was performed, describe the model(s), method(s) to identify the presence and extent of statistical heterogeneity, and software package(s) used.	Line 97-112
	13e	Describe any methods used to explore possible causes of heterogeneity among study results (e.g. subgroup analysis, meta-regression).	Line 118
	13f	Describe any sensitivity analyses conducted to assess robustness of the synthesized results.	Line 121-126
**Reporting bias assessment**	14	Describe any methods used to assess risk of bias due to missing results in a synthesis (arising from reporting biases).	NA
**Certainty assessment**	15	Describe any methods used to assess certainty (or confidence) in the body of evidence for an outcome.	NA
**RESULTS**			
**Study selection**	16a	Describe the results of the search and selection process, from the number of records identified in the search to the number of studies included in the review, ideally using a flow diagram.	Line 128-150 and Figure 1
	16b	Cite studies that might appear to meet the inclusion criteria, but which were excluded, and explain why they were excluded.	Supplementary Material 3
**Study characteristics**	17	Cite each included study and present its characteristics.	Table 1

Topic	No.	Item	Location where item is reported
**Risk of bias in studies**	18	Present assessments of risk of bias for each included study.	Line 147-150 and Table S3 and S4
**Results of individual studies**	19	For all outcomes, present, for each study: (a) summary statistics for each group (where appropriate) and (b) an effect estimate and its precision (e.g. confidence/credible interval), ideally using structured tables or plots.	Figure 2, figure 3, figure 4, supplementary material 2
**Results of syntheses**	20a	For each synthesis, briefly summarise the characteristics and risk of bias among contributing studies.	NA
	20b	Present results of all statistical syntheses conducted. If meta-analysis was done, present for each the summary estimate and its precision (e.g. confidence/credible interval) and measures of statistical heterogeneity. If comparing groups, describe the direction of the effect.	Line 196-210
	20c	Present results of all investigations of possible causes of heterogeneity among study results.	NA
	20d	Present results of all sensitivity analyses conducted to assess the robustness of the synthesized results.	Line 227-244
**Reporting biases**	21	Present assessments of risk of bias due to missing results (arising from reporting biases) for each synthesis assessed.	NA
**Certainty of evidence**	22	Present assessments of certainty (or confidence) in the body of evidence for each outcome assessed.	NA
**DISCUSSION**			
**Discussion**	23a	Provide a general interpretation of the results in the context of other evidence.	Line 265-303
	23b	Discuss any limitations of the evidence included in the review.	Line 309-335
	23c	Discuss any limitations of the review processes used.	Line 309-335
	23d	Discuss implications of the results for practice, policy, and future research.	Line 333-335
**OTHER INFORMATION**			
**Registration and protocol**	24a	Provide registration information for the review, including register name and registration number, or state that the review was not registered.	Line 65-66
	24b	Indicate where the review protocol can be accessed, or state that a protocol was not prepared.	NA
	24c	Describe and explain any amendments to information provided at registration or in the protocol.	NA
**Support**	25	Describe sources of financial or non-financial support for the review, and the role of the funders or sponsors in the review.	Line 364

Topic	No.	Item	Location where item is reported
**Competing interests**	26	Declare any competing interests of review authors.	Line 370
**Availability of data, code and other materials**	27	Report which of the following are publicly available and where they can be found: template data collection forms; data extracted from included studies; data used for all analyses; analytic code; any other materials used in the review.	Line 367-369

Table S1. PRISMA 2020 Main Checklist

Topic	No.	Item	Reported?
**TITLE**			
**Title**	1	Identify the report as a systematic review.	Yes
**BACKGROUND**			
**Objectives**	2	Provide an explicit statement of the main objective(s) or question(s) the review addresses.	Yes
**METHODS**			
**Eligibility criteria**	3	Specify the inclusion and exclusion criteria for the review.	Yes
**Information sources**	4	Specify the information sources (e.g. databases, registers) used to identify studies and the date when each was last searched.	Yes
**Risk of bias**	5	Specify the methods used to assess risk of bias in the included studies.	Yes
**Synthesis of results**	6	Specify the methods used to present and synthesize results.	Yes
**RESULTS**			
**Included studies**	7	Give the total number of included studies and participants and summarise relevant characteristics of studies.	Yes
**Synthesis of results**	8	Present results for main outcomes, preferably indicating the number of included studies and participants for each. If meta-analysis was done, report the summary estimate and confidence/credible interval. If comparing groups, indicate the direction of the effect (i.e. which group is favoured).	Yes
**DISCUSSION**			
**Limitations of evidence**	9	Provide a brief summary of the limitations of the evidence included in the review (e.g. study risk of bias, inconsistency and imprecision).	No
**Interpretation**	10	Provide a general interpretation of the results and important implications.	Yes
**OTHER**			
**Funding**	11	Specify the primary source of funding for the review.	No
**Registration**	12	Provide the register name and registration number.	Yes

Table S2. PRISMA Abstract Checklist

Author	Aim of the study	Inclusion of consecutive patients	Prospective collection of data	Endpoint appropriate to the study aim	Unbiased evaluation of endpoints	Follow up period appropriate to the major endpoint	Loss to follow up not exceeding 5%	Sample calculation	Score
**Bardi et al. (2021)**	2 Reported and adequate	2 Reported and adequate	1* No protocol registration	2 Reported and adequate	2 Reported and adequate	2 Reported and adequate	2 Reported and adequate	0 Not reported	13/16
**Blonz et al. (2021)**	2 Reported and adequate	2 Reported and adequate	1* No protocol registration	2 Reported and adequate	2 Reported and adequate	1* Insufficient length	2 Reported and adequate	0 Not reported	12/16
**Buetti et al. (2020)**	2 Reported and adequate	2 Reported and adequate	1* No protocol registration	2 Reported and adequate	2 Reported and adequate	2 Reported and adequate	2 Reported and adequate	0 Not reported	13/16
**Gamberini et al. (2020)**	2 Reported and adequate	2 Reported and adequate	2 Reported and adequate	2 Reported and adequate	2 Reported and adequate	2 Reported and adequate	2 Reported and adequate	0 Not reported	14/16
**Garcia-Vidal et al. (2020)**	2 Reported and adequate	2 Reported and adequate	1* No protocol registration	2 Reported and adequate	2 Reported and adequate	2 Reported and adequate	2 Reported and adequate	0 Not reported	13/16
**Giacobbe et al. (2021)**	2 Reported and adequate	2 Reported and adequate	1* No protocol registration	2 Reported and adequate	2 Reported and adequate	2 Reported and adequate	2 Reported and adequate	2 Reported and adequate	15/16

**Grasselli et al. (2021)**	2 Reported and adequate	2 Reported and adequate	2 Reported and adequate	2 Reported and adequate	2 Reported and adequate	2 Reported and adequate	2 Reported and adequate	0 Not reported	14/16
**Llitjos et al. (2021)**	2 Reported and adequate	2 Reported and adequate	1* No protocol registration	2 Reported and adequate	2 Reported and adequate	2 Reported and adequate	2 Reported and adequate	0 Not reported	13/16§
**Luyt et al. (2020)**	2 Reported and adequate	2 Reported and adequate	1* No protocol registration	2 Reported and adequate	2 Reported and adequate	2 Reported and adequate	2 Reported and adequate	0 Not reported	13/16
**Maes et al. (2021)**	2 Reported and adequate	2 Reported and adequate	1* No protocol registration	2 Reported and adequate	2 Reported and adequate	2 Reported and adequate	2 Reported and adequate	0 Not reported	13/16
**Moretti et al. (2021)**	2 Reported and adequate	2 Reported and adequate	1* No protocol registration	2 Reported and adequate	2 Reported and adequate	2 Reported and adequate	2 Reported and adequate	0 Not reported	13/16
**Pederson et al. (2020)**	2 Reported and adequate	1* Unclear description of inclusion process and study period	1* No protocol registration	1* No pre-specified outcomes	2 Reported and adequate	1* Insufficient length	2 Reported and adequate	0 Not reported	10/16
**Razazi et al. (2020)**	2 Reported and	2 Reported and adequate	1* No protocol registration	2 Reported and adequate	2 Reported and	2 Reported and adequate	2 Reported and adequate	0 Not reported	13/16

	adequate				adequate				
**Rouzè et al. (2021)**	2 Reported and adequate	2 Reported and adequate	2 Reported and adequate	2 Reported and adequate	2 Reported and adequate	2 Reported and adequate	2 Reported and adequate	0 Not reported	14/16
**Schmidt et al. (2020)**	2 Reported and adequate	2 Reported and adequate	1* No protocol registration	2 Reported and adequate	2 Reported and adequate	2 Reported and adequate	2 Reported and adequate	0 Not reported	13/16
**Sharov et al. (2020)**	2 Reported and adequate	1* Unclear description of inclusion process and study period	1* No protocol registration	2 Reported and adequate	2 Reported and adequate	2 Reported and adequate	2 Reported and adequate	0 Not reported	12/16
**Søgaard et al. (2021)**	2 Reported and adequate	2 Reported and adequate	1* No protocol registration	2 Reported and adequate	2 Reported and adequate	2 Reported and adequate	2 Reported and adequate	0 Not reported	13/16
**Souarez de la Rica (2021)**	2 Reported and adequate	2 Reported and adequate	1* No protocol registration	2 Reported and adequate	2 Reported and adequate	2 Reported and adequate	2 Reported and adequate	0 Not reported	13/16
**Tanaka et al. (2020)**	2 Reported and adequate	2 Reported and adequate	1* No protocol registration	2 Reported and adequate	2 Reported and adequate	2 Reported and adequate	2 Reported and adequate	0 Not reported	13/16
**Zhou et al. (2020)**	2 Reported	2 Reported and	1* No protocol	2 Reported and	2 Reported	2 Reported and	2 Reported and	0 Not	13/16

and adequate	adequate	registration	adequate	and adequate	adequate	adequate	reported

Table S3. Quality assessment of studies according to MINORS score for non-comparative studies.

Qualitative assessment was performed using the MINORS score. The eight items are scored 0 (not reported), 1 (reported but inadequate) or 2 (reported and adequate). The global ideal score being 16 for non-comparative studies.

* For the domains judged as 'reported but inadequate', the table provides a brief explanation of the detected reason for inadequacy.

§ Please also see the additional score for comparative studies at Table S4

	**An adequate control group**	**Contemporary groups**	**Baseline equivalence of groups**	**Adequate statistical analyses**	**Additional score for comparative studies**
**Llitjos et al. (2021)**	2 Reported and adequate	1* Historical cohort	1* Potential confounders among baseline characteristics	2 Reported and adequate	6/8
**Luyt et al. (2020)**	2 Reported and adequate	1* Historical cohort	1* Potential confounders among baseline characteristics	1* Unadjusted analysis	5/8
**Maes et al. (2021)**	2 Reported and adequate	2 Reported and adequate	1* Potential confounders among baseline characteristics	2 Reported and adequate	7/8

**Razazi et al. (2020)**	2<br>Reported and adequate	2<br>Reported and adequate	1*<br>Potential confounders among baseline characteristics	2<br>Reported and adequate	7/8
**Rouzè et al. (2021)**	2<br>Reported and adequate	2<br>Reported and adequate	1*<br>Potential confounders among baseline characteristics	2<br>Reported and adequate	7/8

Table S4. Quality assessment of studies according to MINORS additional score for comparative studies.

Qualitative assessment was performed using the MINORS score. The four items are scored 0 (not reported), 1 (reported but inadequate) or 2 (reported and adequate). The global ideal score being 24 (sum of non-comparative and comparative study items) for comparative studies.

* For the domains judged as 'reported but inadequate', the table provides a brief explanation of the detected reason for inadequacy.
